# The impact of acute and chronic aerobic and resistance exercise on stem cell mobilization: A review of effects in healthy and diseased individuals across different age groups

**DOI:** 10.1016/j.reth.2024.04.013

**Published:** 2024-05-07

**Authors:** Wei Li, Lingzhen Chen, S. Mohammad Sajadi, Sh. Baghaei, Soheil Salahshour

**Affiliations:** a Department of Sports Medicine, Fourth Medical Center of PLA General Hospital, Beijing 100048, China; b Department of Sports and Arts, Zhejiang Gongshang University HangZhou College of Commerce, No. 66, South Huancheng Road, Tonglu, Hangzhou, China; c Department of Nutrition, Cihan University-Erbil, Kurdistan Region, Iraq; d Department of Mechanical Engineering, Khomeinishahr Branch, Islamic Azad University, Iran; e Faculty of Engineering and Natural Sciences, Istanbul Okan University, Istanbul, Turkey; f Faculty of Engineering and Natural Sciences, Bahcesehir University, Istanbul, Turkey; g Department of Computer Science and Mathematics, Lebanese American University, Beirut, Lebanon

**Keywords:** Exercise, Stem cells, Cellular mobilization, Progenitor cells, CD34^+^, Sport management

## Abstract

Stem cells (SCs) play a crucial role in tissue repair, regeneration, and maintaining physiological homeostasis. Exercise mobilizes and enhances the function of SCs. This review examines the effects of acute and chronic aerobic and resistance exercise on the population of SCs in healthy and diseased individuals across different age groups. Both acute intense exercise and moderate regular training increase circulating precursor cells CD34^+^ and, in particular, the subset of angiogenic progenitor cells (APCs) CD34+/KDR+. Conversely, chronic exercise training has conflicting effects on circulating CD34^+^ cells and their function, which are likely influenced by exercise dosage, the health status of the participants, and the methodologies employed. While acute activity promotes transient mobilization, regular exercise often leads to an increased number of progenitors and more sustainable functionality. Short interventions lasting 10–21 days mobilize CD34+/KDR + APCs in sedentary elderly individuals, indicating the inherent capacity of the body to rapidly activate tissue-reparative SCs during activity. However, further investigation is needed to determine the optimal exercise regimens for enhancing SC mobilization, elucidating the underlying mechanisms, and establishing functional benefits for health and disease prevention. Current evidence supports the integration of intense exercise with chronic training in exercise protocols aimed at activating the inherent regenerative potential through SC mobilization. The physical activity promotes endogenous repair processes, and research on exercise protocols that effectively mobilize SCs can provide innovative guidelines designed for lifelong tissue regeneration. An artificial neural network (ANN) was developed to estimate the effects of modifying elderly individuals and implementing chronic resistance exercise on stem cell mobilization and its impact on individuals and exercise. The network's predictions were validated using linear regression and found to be acceptable compared to experimental results.

## 1 Introduction

**Fig. 1:**
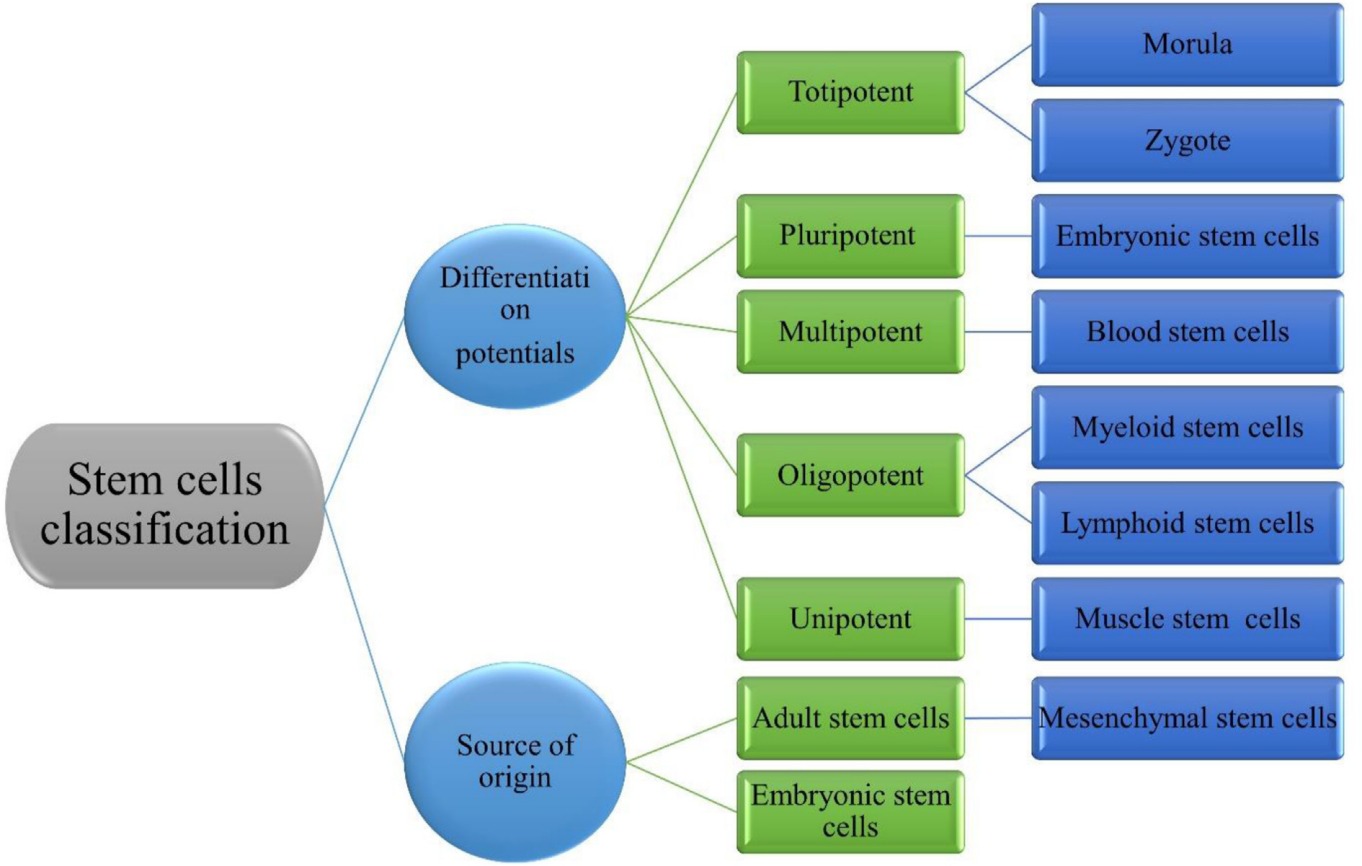
Stem cell classification based on potency and origin illustrated.

Stem cells (SCs) are a population of cells capable of differentiation into various cell types and proliferating through mitotic divisions to maintain population size. Based on their differentiation potential, SCs are classified into totipotent (capable of differentiating into all cell types), pluripotent (able to differentiate into cells of the embryonic trophoblast lineage), multipotent (able to differentiate into various cell types within a specific lineage), and unipotent (able to differentiate into a single type of cell) [1,2]. One of the pluripotent SC types includes embryonic SCs (ESCs). Hematopoietic SCs (HSCs), residing within the bone marrow, are considered a multipotent SC population and satellite cells in skeletal muscle are among the examples of unipotent SCs [[1], [2], [3]]. In addition to classifying SCs based on their differentiation potential, they can also be categorized based on their origin, including adult SCs (ASCs), ESCs, and umbilical cord SCs. Fig. 1 illustrates the classification of SCs based on their potency and origin. ESCs are among the pluripotent SCs that are extracted from 3-5-day-old blastocyst embryos and are found in the inner cell mass before implantation [[3], [4], [5], [6]]. Umbilical cord SCs are extracted from the umbilical cord of the fetus. This category of SCs has a larger volume and number compared to other SCs and also has easier accessibility. ASCs are more specialized cells and are present in various adult tissues. These cells play a crucial role in tissue homeostasis and remain in a quiescent state as long as the body does not require them. A limited number of these cells exist in most tissues and act as an endogenous repair system in response to injury or damage [[7], [8], [9]]. Neural SCs (NSCs), HSCs, mesenchymal SCs (MSCs), and skin SCs (SSCs) are examples of ASCs. According to research, NSCs are capable of differentiating into neurons, oligodendrocytes, and astrocytes. HSCs can differentiate into mature blood cells, mainly found in the bone marrow. MSCs possess the capacity to differentiate into ectodermal or endodermal lineage cells, bone, adipose tissue, and cartilage [[8], [9], [10]]. These cells naturally reside within tissues such as placenta tissues, adipose tissue, bone marrow, and peripheral blood. Additionally, SSCs demonstrate the capability to differentiate into various cell types, including cutaneous and hair follicle cells. The therapeutic objective of utilizing SCs involves enhancing patients' quality of life, mitigating chronic diseases, and establishing novel approaches for the treatment of diseases and tissue injuries [3,[10], [11], [12]]. SCs have broad applications in the field of medicine, including tissue regeneration, disease modeling, and more. Table 1 provides an overview of various applications, types of SCs involved, and the primary sources from which these SC are derived. SC therapies can be administered in autologous, allogenic, or xenogeneic modalities. Autologous therapy involves the utilization of an individual's SCs or their secretions, which presents fewer concerns regarding compatibility and immunogenicity. Allogenic therapy employs SCs from a healthy donor of the same species, which still necessitates precise evaluation to prevent immunogenic reactions [[13], [14], [15], [16]]. In xenogeneic approaches, the donor and recipient SCs originate from different species. In this method, the use of human SCs in animal models has garnered significant attention for assessing their performance within the animal body. However, such invasive techniques employing SCs, such as bone marrow aspiration, raise considerable concerns and challenges [[15], [16], [17]]. Another non-invasive method for utilizing bone marrow-derived SCs involves their liberation into the peripheral circulation during therapies, stimulated through pharmacological agents and physical activities. This article examines the influence of exercise on SC mobilization, investigating its effects during acute exercise periods and short-to long-term training sessions. In the field of medical regeneration, various pharmacological strategies have been investigated to enhance the production and capabilities of SCs. Table 2 presents some of the drugs used to increase SCs, their associated SC types, mechanisms of action, as well as the advantages and disadvantages involved. The results offer valuable insights into the correlation between physical activity and SC release [[17], [18], [19], [20]]. This review aims to investigate the effects of acute and chronic aerobic and resistance exercise on stem cell (SC) populations in healthy and diseased individuals across various age groups. It focuses on the impact of exercise on circulating precursor cells, particularly angiogenic progenitor cells (APCs), while considering exercise dosage, participant health status, and methodological variations. Additionally, the review identifies gaps in current knowledge and underscores the need for further research to determine optimal exercise regimens that enhance SC mobilization, elucidate underlying mechanisms, and establish the functional benefits for both health promotion and disease prevention [[18], [19], [20], [21], [22]]. The objective of this comprehensive review is to examine the effects of acute and chronic aerobic and resistance exercise on stem cell mobilization in healthy and diseased individuals across different age groups. By synthesizing existing literature, identifying knowledge gaps, and proposing future research directions, this review contributes to the field by providing a thorough analysis of both acute and chronic exercise effects on stem cell populations, encompassing aerobic and resistance exercise modalities. It highlights the significance of considering age groups and the health status of individuals in understanding the impact of exercise on stem cells. Moreover, the integration of an artificial neural network (ANN) for predicting the effects of modifying elderly individuals and implementing chronic resistance exercise on stem cell mobilization represents a novel approach. The review emphasizes the need for further investigation, standardized methodologies, and exploration of exercise-induced stem cell mobilization in therapeutic applications. Notably, the review presents novel insights by comprehensively examining the mobilization and function of precursor cells, including APCs, in response to acute intense exercise and regular training, thereby shedding light on the factors influencing SC mobilization. Importantly, it emphasizes the importance of future investigations to explore exercise protocols that effectively mobilize SCs, investigate underlying mechanisms, and ascertain the functional advantages derived from exercise-induced SC activation, thus offering innovative guidelines for integrating exercise as a strategic approach to harness the regenerative potential of SCs for tissue repair and maintenance. In this study, a shallow progressive artificial neural network (SPANN) with a hidden layer was developed to explore a wider range of variables, including stem cell mobilization, the effects on individuals, and the impact on exercise among elderly individuals. The neural network inputs consisted of predicted patient data and chronic resistance exercise. Furthermore, the performance of the ANN was evaluated using linear regression. The predicted outcomes generated by the neural network were reported, and the estimation process was assessed.

**Table 1:** Applications of SCs in medicine.

Application	Description	SC Type	Supply Sources
Tissue regeneration	Repair damaged tissues like heart, brain, skin	ASCs, ESCs	Bone marrow, adipose tissue, umbilical cord
Cell therapy	Treat diabetes, neurological disorders, spinal injuries	ASCs, induced pluripotent SCs	Peripheral blood, bone marrow, adipose tissue
Drug testing	Test drug safety and efficacy	ESCs, induced pluripotent SCs	Embryos, reprogrammed adult cells
Disease modeling	Model diseases like cancer, genetic disorders	Induced pluripotent SCs	Patient-derived cells reprogramed
Cord blood banking	For future transplant and therapy	HSCs	Umbilical cord blood
Tissue engineering	Grow new organs/tissues	ESCs, ASCs	Embryos, bone marrow, fat tissue
Gene therapy	Deliver gene therapy	HSCs	Bone marrow, peripheral blood
Cancer research	Study tumor development	Cancer SCs	Patient tumor samples
Toxicity testing	Assess drug/toxin toxicity	ESCs, iPSCs	Embryos, reprogrammed cells

**Table 2:** Some drugs used to increase the number of SCs.

Drug	SC Type	Mechanism of Action	Advantages	Disadvantages
G-CSF	HSCs	Binds to HSC receptors, stimulating proliferation and differentiation	Increased circulating HSCs Improved CV function Reduced inflammation	Bone pain Spleen enlargement Potential over mobilization of leukocytes
Plerixafor	HSCs	Blocks CXCR4 to aid HSC release from bone marrow	Rapid, robust HSC mobilization Fewer side effects compared to GCSF	Expensive Potential for leukemic cell mobilization
Lithium	NSCs	Increases NSC proliferation and self-renewal	Increased neurogenesis Potential cognitive benefits	Long-term use toxicity Unclear optimal dosing
Metformin	Endothelial progenitor cells (EPC)	Activates AMPK pathway	Increased EPC number and function Improved vascular health	Common but mild gastrointestinal side effects
Resveratrol	NSCs	Activates SIRT1 pathway	Increased NSC proliferation in animal studies	Limited human evidence Optimal dose unknown
Quercetin	HSCs	Mechanism not fully understood	Safe, inexpensive flavonoid Enhanced migration and proliferation of HSCs	Requires further study to confirm effects
Tadalafil	EPCs	Inhibits PDE5 to increase cGMP levels	Increased EPC mobilization Potential improvement in erectile dysfunction	Headaches Back pain Nasal congestion
Darbepoetin α	Erythroid progenitor cells	Stimulates erythropoiesis like endogenous erythropoietin	Treats anemia Increased red blood cell production	Increased risk of stroke, thrombosis, and CV events

## 2 Stimulation of SC production through exercise

There are several key mechanisms through which exercise stimulates increased collagen production in the skin. These mechanisms include increased blood flow and nutrient delivery, release of growth factors, and stress and tissue damage [[25], [26], [27], [28]]. Increased blood flow and nutrient delivery during exercise improve circulation throughout the body, including the skin. This increased blood flow delivers more oxygen and nutrients like amino acids necessary for collagen synthesis while removing waste products. It promotes optimal conditions for collagen production and repair of existing collagen fibers [[29], [30], [31], [32], [33]]. The release of growth factors is another mechanism stimulated by exercise. When muscles contract during exercise, they release myokines, which are growth factors and cytokines that circulate in the body. These myokines stimulate fibroblasts responsible for collagen synthesis in the skin, with IGF-1 playing a key role in upregulating collagen gene expression and production. Exercise also induces stress and tissue damage to muscles and connective tissues [[34], [35], [36]]. This stress and micro-injuries stimulate an inflammatory response and repair adaptation and remodeling processes [[37], [38], [39], [40], [41]]. As a result, damaged collagen fibers are repaired and replaced, leading to the deposition of new collagen. Over time, this process leads to the development of thicker and stronger collagen bundles. Additionally, regular exercise helps reduce cortisol levels [42,43]. While stress hormones like cortisol are naturally elevated during exercise, chronic high levels can actually break down collagen in the skin. Regular exercise helps lower resting cortisol levels, supporting collagen maintenance and synthesis.

### 2.1 Acute exercise

**Fig. 2:**
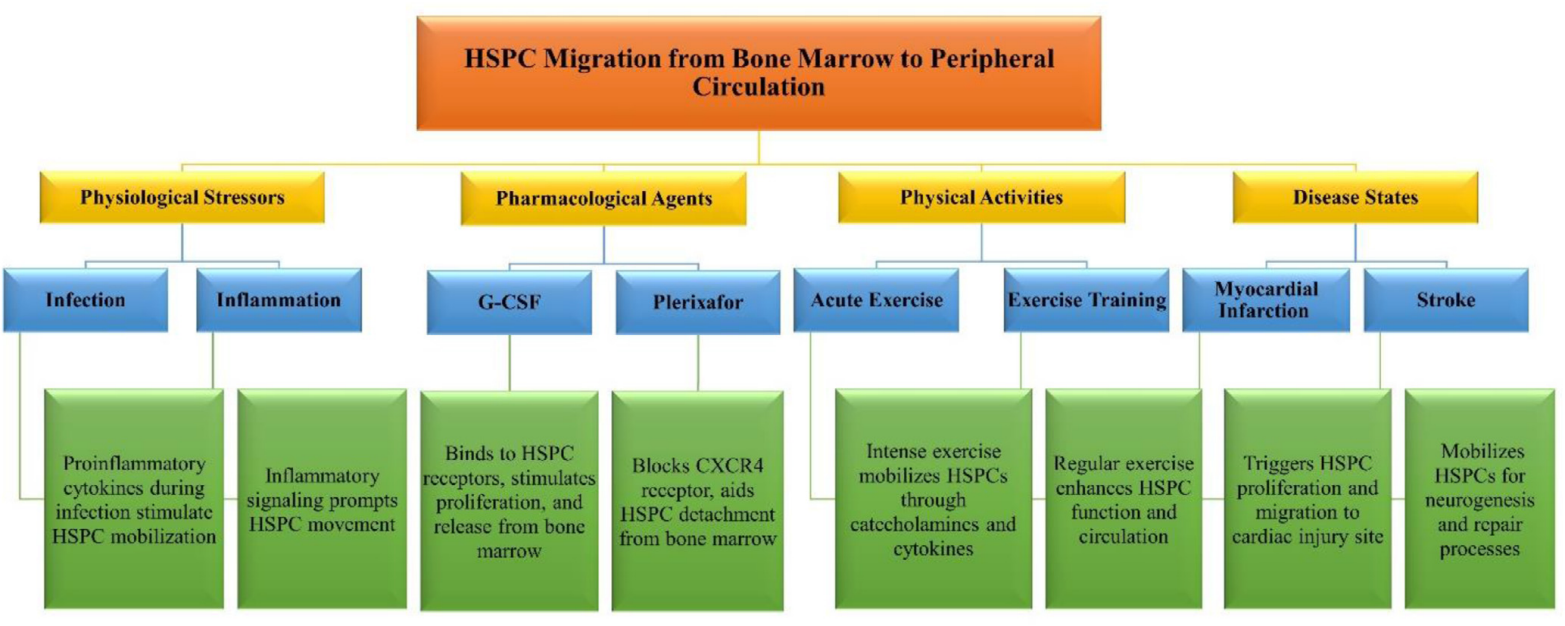
Factors and conditions stimulating HSPC migration from the bone marrow to the peripheral blood.

**Fig. 3:**
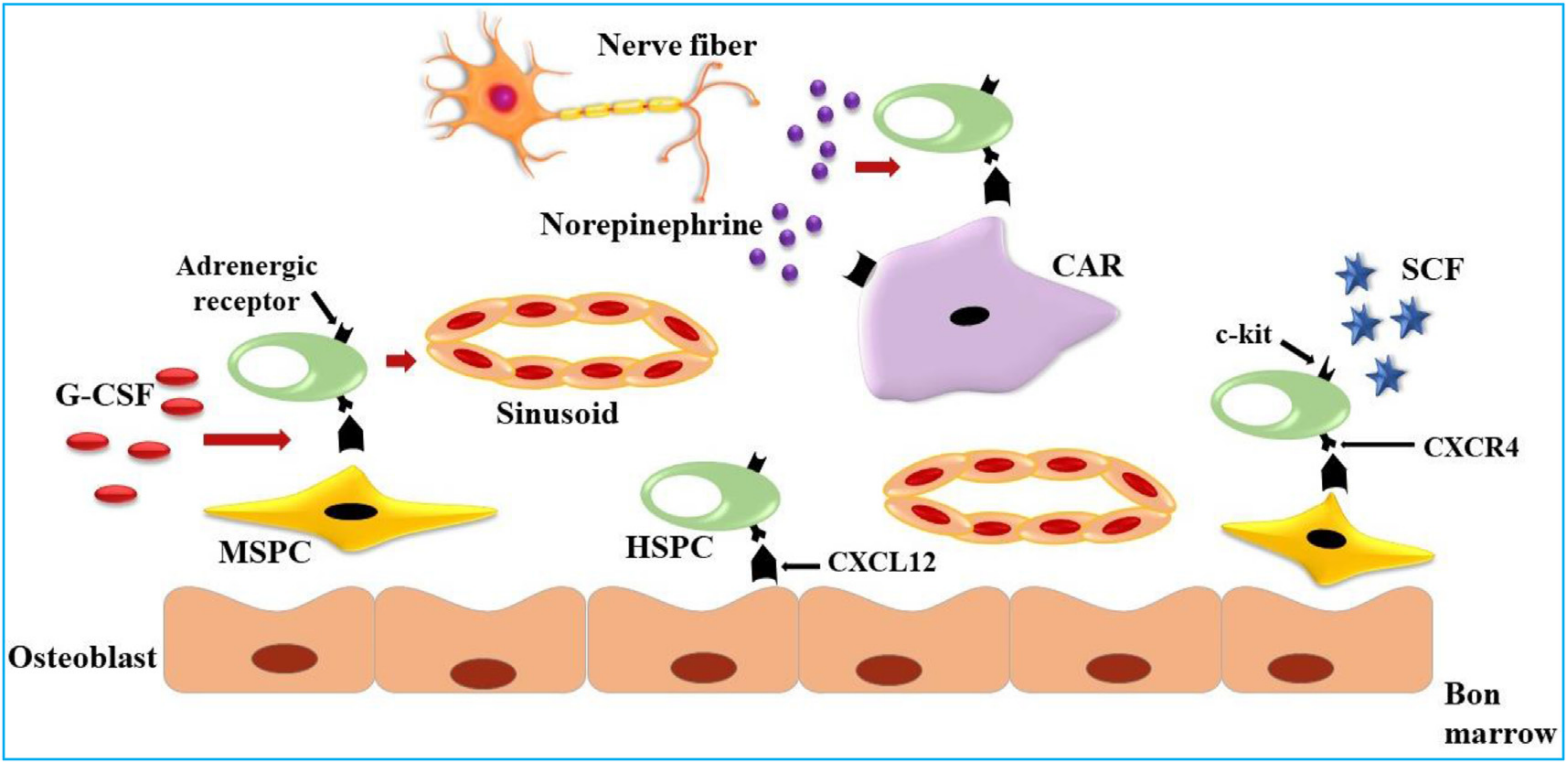
The process of HSC mobilization from the bone marrow into the peripheral bloodstream due to exercise.

Typically, a small number of hematopoietic stem and progenitor cells (HSPCs) reside in the bone marrow during homeostasis. However, under certain conditions such as physiological stress (infection, inflammation, or exercise), the migration of HSPCs from the bone marrow to the peripheral circulation is stimulated, resulting in an increased quantity of HSPCs. Fig. 2 shows the factors and conditions that stimulate the migration of HSPCs from the bone marrow to the peripheral blood [[20], [21], [22]]. These cells, characterized by the expression of CD34^+^ and other cell surface markers, primarily reside in specialized niches or compartments within the bone marrow. Upon stimulation and augmentation of these cells, they can relocate to distant sites or return to the bone marrow for potential utilization in tissue repair or regeneration [[21], [22], [23]]. CD34^+^ progenitor cells (PCs) play a significant role as potent precursors capable of engrafting in the patient's bone marrow [24]. Fig. 3 illustrates the process of HSC mobilization from the bone marrow into the peripheral bloodstream during exercise. Acute bouts of exercise can stimulate the movement of HSPCs from their niche environments in the bone marrow into peripheral blood circulation. Within the bone marrow, HSPCs receive signals from surrounding cells such as osteoblasts, MSCs, CXCL12-abundant reticular (CAR) cells, and mature blood cells. These interactions serve to maintain SC quiescence and self-renewal under baseline conditions. During exercise, the contraction of muscles leads to the release of cytokines and other signaling molecules which act to detach HSPCs from their niches. Factors such as stem cell factor (SCF), granulocyte colony stimulating factor (G-CSF), and norepinephrine facilitate the entry of these cells into sinusoidal vessels and increase their numbers in the circulating blood [[25], [26], [27]]. The first investigations on the mobilization of precursor cells resulting from exercise were conducted by Brightman et al. [28]. According to the measurements of colony-forming unity-granulocyte macrophage (CFU-GM), the highest concentration of these cells was observed immediately after exercise, and in sedentary individuals, the concentration of precursor cells returned to baseline after 60 min of exercise. Within the 15-min time frame following exercise activities, paradigms such as the “All-Out” rowing test [29] and the maximal ergometer cycle test [30], with the identification of phenotypic markers for HSPCs, reported a twofold increase in circulating CD34^+^ cell levels. Another study conducted by Laufs et al. [31] aimed to determine the effects of physical activity on specific subsets of CD34^+^ cells in healthy young adults. They reported that 30 min of high-intensity or moderate-intensity running (82% and 68% VO_2max_, respectively) resulted in an increase in the number of CD34+/KDR+ and CD34+/CD133+ cells within 10 min after exercise, with peak values observed between 10 and 30 min. Conversely, running for a 10-min duration had no significant effect on this CD34^+^ population [31]. Subsequent studies involving continuous cycling for up to 4 h at a 70% ventilatory threshold demonstrated time-dependent increases in CD34^+^ and CD34+/KDR + cells in circulation, with the highest levels observed 3–4 h after exercise, gradually decreasing in the subsequent hours, with the greatest decrease occurring at 30 min post-exercise. Vascular endothelial growth factor (VEGF) and interleukin-6, believed to act as chemokines released from active muscles, are implicated as the primary and subsequent regulators of these types of cells in the blood [32]. Furthermore, Kroepfl et al. [33] utilized incremental cycling exercise to depict the kinetics of CD34^+^ and 45^dim^-expressing precursor cells in peripheral blood and observed higher levels within 10 min post-exercise, which then declined to baseline after 30 min of exercise [33,34].

Other studies have examined the effects of acute exercise on the CD34^+^ cell population in both older and younger individuals. Notable mobilization of CD34+/CD4^dim^ and CD34+/CD45^dim^/KDR cells occurred in both age groups, although the absolute response was less pronounced in older individuals [35,36]. However, relative changes in CD34^+^ cell numbers after exercise, when calculated as a percentage of baseline diversity, did not differ significantly between young adults and older adults. Further research demonstrated that the number of CD34+/KDR + cells increase below the maximal level 30 min after a treadmill session in older adults, without affecting the overall CD34^+^ level [37]. In contrast, no changes in CD34+/KDR+ were observed in young men and women following submaximal cycling. Therefore, even submaximal exercise intensity/duration appears to be crucial for inducing cellular changes in CD34^+^ [38]. One study reported immediate mobilization of CD34^+^ following intense cycling but not low-effort exercise in physically active young men [39]. These reports indicate that submaximal activity may elicit migration of CD34^+^ subsets if sufficiently challenging, and high-impact methods may stimulate faster responses in CD34^+^ compared to low-impact choices such as cycling. Based on the conducted research, it appears that acute maximal exercise increases the number of CD34^+^ cells. Baker et al. [39] reported a significant increase in circulating CD34+/KDR + cells in both young and older men following a high-intensity cycling race, contrasting with a longer submaximal trial [30,32]. Similarly, comprehensive rowing for 3–4 min doubled the CD34^+^ count in trained athletes [36]. Other studies demonstrated that maximal treadmill running or cycling races resulted in increased levels of CD34+/KDR + after exercise [40,41]. These reports indicate that maximal exercise mobilizes CD34^+^ populations more effectively than submaximal protocols. It should be noted that the responses of CD34^+^ subsets to maximal activity vary among different studies, likely due to variations in modulation or cell detection methods. Generally, the evidence suggests that high-intensity exercise serves as a stronger stimulus for CD34^+^ cell mobilization compared to low to moderate intensity protocols [[42], [43], [44], [45], [46]]. The responses of CD34^+^ subsets to maximal activity differ between studies, which may be attributed to differences in methodology or cell detection techniques. In conclusion, the evidence suggests that exercise with high activity serves as a stronger stimulus for CD34^+^ cell migration compared to low to moderate-intensity activities [45,[47], [48], [49], [50], [51]]. In addition to increasing the number of CD34^+^ cells, acute exercise can impact the function of these cells [[52], [53], [54], [55], [56]]. A study analyzed the levels of intracellular nitric oxide (NO) and superoxide (O_2_^−^) in CD34^+^ and CD34-cells from sedentary individuals and endurance-trained individuals before and after submaximal cycling [57]. Initially, CD34^+^ cells from sedentary individuals demonstrated higher levels of NO and O_2_^−^ compared to endurance-trained individuals, indicating increased oxidative stress. However, these differences disappeared after acute exercise, likely due to a reduction in nicotinamide adenine dinucleotide phosphate (NADPH) oxidase activity, suggesting that regular exercise may decrease oxidative stress in CD34^+^ cells. Other results from the study by Kroepfl et al. [33] demonstrate that while CD34+/CD45^dim^ cells increase after maximal cycling, their replicative capacity decreases, potentially due to exercise-induced oxidative stress. A subsequent experiment attributed the increase in CD34+/CD45^dim^ cells following exercise to an associated increase in noradrenaline, which reduced their functional capacity under laboratory conditions [34]. Further research is needed to elucidate the duration of the decline circulating angiogenic cells (CAC) function after exercise and whether regular exercise can mitigate this acute response.

### 2.2 Long-term exercise training

A significant body of research has consistently demonstrated that engaging in regular aerobic exercise is strongly correlated with favorable alterations in circulating lipid profiles. These alterations encompass reductions in triglyceride levels, total cholesterol, and LDL cholesterol, alongside elevations in HDL cholesterol levels [[58], [59], [60], [61]]. Moreover, aerobic exercise has been consistently associated with decreased circulating markers associated with oxidative stress and systemic inflammation, including 8-isoprostane, interleukin-6, and C-reactive protein. Continuous aerobic exercise enhances the endogenous antioxidant defense capacity, including increased activity of enzymes such as O_2_^−^ dismutase and glutathione peroxidase [[62], [63], [64], [65], [66]]. These beneficial effects of routine aerobic exercise all contribute to the enhanced biological availability of NO. In addition to these systemic effects, studies consistently demonstrate that engaging in regular aerobic activities is associated with an increase in the number of circulating CD34^+^ precursor cells, both in healthy populations and individuals with chronic conditions such as heart failure or diabetes [45,[67], [68], [69]]. This exercise-induced increase in CD34^+^ cells occur regardless of age range. The elevation of CD34^+^ cells is generally accompanied by improved endothelial function, likely due to the role of these precursor cells in vascular repair and angiogenesis [[69], [70], [71], [72], [73]]. Regular aerobic exercise provides significant benefits in terms of circulating biomarkers, antioxidant capacity, precursor cell count, and endothelial function in adults of all ages [74,75]. While several studies have demonstrated an augmentation in both the quantity and functionality of CD34^+^ cells as a result of aerobic exercises, the results are not consistently reproducible across all exercise regimens. Thijssen et al. [36,82] conducted a study that revealed no discernible effects on the baseline or maximum mobilization of CD34^+^ or CD34+/KDR + cells in older men following an 8-week program of moderate-intensity cycling, comprising 20-min sessions conducted three times per week. This indicates that shorter and more intense aerobic exercise may not provide adequate stimulation for precursor cell mobilization. Furthermore, no changes in the quantity of CD34+/KDR+/CD45dim cells were observed after 12 weeks of either intermittent or continuous aerobic exercise in patients diagnosed with coronary artery disease (CAD) [76,77]. The lack of change observed in this study could potentially be attributed to the participants' disease status, as individuals with CAD tend to exhibit impaired precursor cell function. Taking a broader perspective beyond structured exercise interventions, when comparing precursor cell counts cross-sectionally between sedentary populations and endurance exercise, diversity has also been examined. Generally, while structured aerobic exercise often increases the population and cellular function of CD34^+^ cells, different results are also found in some studies. Variations in program variables along with subject characteristics (intensity, age, duration, exercise mode, disease classification) and cellular analysis methods likely contribute to conflicting results. Advanced age is typically associated with lower circulating CD34^+^ cells [[78], [79], [80], [81], [82]]. Research conducted by Witkowski et al. [74] on sedentary older individuals and professional athletes did not report any differences in CD34^+^ or CD34+/KDR + cells. However, after a 10-day exercise cessation period, the number of CD34^+^ cells decreased in the older athletes. Since aging independently increases the risk of cardiovascular diseases) CVD(, further research on suitable exercise regimens for older individuals is necessary, which may improve the number of CD34^+^ cells [[82], [83], [84], [85]]. This indicates that even transient increases in precursor cells may be accompanied by sustainable functional achievements and protection of the heart and vasculature. Therefore, structured programs for generating CD34^+^ cells and necessary functional adaptations are not required [85].

### 2.3 Short-term exercise training

Recent studies have demonstrated that short-term exercise can have a significant impact on SCs in the body. Mechanical stress and impact during exercise activate satellite cells. According to research, a single exercise session rapidly mobilizes satellite cells, leading to their proliferation and an increase in the body's capacity for repair and regeneration [24,[86], [87], [88], [89], [90], [91]]. Furthermore, exercise stimulates the secretion of growth factors and cytokines such as Interleukin-6 (IL-6), IL-1, and G-CSF, which regulate the function of satellite cells. According to conducted studies, growth factors such as IGF-1 and HGF increase within 30 min after the initiation of exercise [[92], [93], [94]]. Table 3 illustrates the effects of exercise-induced growth factors on ACSs. During exercise, released growth factors act through various mechanisms to stimulate the proliferation, migration, and differentiation of ACSs. As mentioned, other populations of SCs (such as NSC, endothelial, and HSCs) also respond to acute exercise. The activation and rapid proliferation of SCs during short-term exercise highlight the body's inherent capacity to respond and adapt to physical activity stimuli. In a study, the effects of a 10-day aerobic exercise program (60 min at 70% VO_2max_) were examined in older adults who were previously sedentary [[95], [96], [97], [98], [99]]. According to the results, there was no significant increase in the total number of CD34^+^ cells, but an increase in the number of CD34+/KDR+ and KDR + cells was observed [100].

**Table 3:** Effects of exercise-induced growth factors on ASCs.

Growth Factor	Source Tissue	SC Target	Mechanism of Action	Effect on SCs
VEGF	Skeletal muscle	EPCs	Promotes cell mobility and proliferation	Increased migration and proliferation
HGF	Skeletal muscle	HSCs	Mobilizes cells from bone marrow	Bone marrow mobilization
SDF-1α	Skeletal muscle	CXCR4+ SCs	Induces mobilization from bone marrow	Stem cell mobilization from bone marrow
BDNF	Hippocampus	NSCs	Increases cell proliferation	Increased hippocampal neurogenesis
IGF-1	Liver, skeletal muscle	Satellite cells	Activates and induces proliferation	Muscle satellite cell activation and proliferation
FGF-2	Skeletal muscle	EPCs	Recruits' cells for angiogenesis	Angiogenesis through EPC recruitment
TGF-β	Platelets	HSCs	Regulates quiescence in niche	Maintenance of bone marrow quiescence
G-CSF	Immune cells	HSCs	Induces mobilization from bone marrow	Bone marrow SC mobilization

These results are consistent with the study of Gatta et al. [101], that a supervised 3-week aerobic exercise program (30 min twice a day at 75–85% VO_2max_) increased the CD34+/KDR + cells in elderly patients with heart failure. In another study involving young male cyclists, a 3-week moderate exercise program (45–60 min at 50–70% VO_2max_) was investigated. This program resulted in a 285% increase in CD34+/KDR + cellular levels. However, a different study examining the mobilization of CD34^+^ cells through exercise yielded diverse results. A 6-week intervention comparing aerobic, resistance, and combined exercises in moderately active young men showed no significant effect on the number of CD34^+^ cells between the groups, despite improvements in strength. Furthermore, the number of CD34^+^ cells remained unchanged compared to baseline after a 3-week follow-up period [102]. Rakobowchuk et al. [103] did not observe any changes in CD34^+^ concentration after a 6-week high-intensity interval training or moderate continuous aerobic exercise in healthy young individuals, although significant inter-individual variability was observed. The lack of mobilization among individuals with cardiovascular (CV) fitness suggests that the response of PCs may be dependent on baseline performance. A multitude of studies conducted in the field of biological and medical research have yielded significant results, expanding our understanding of numerous facets of these disciplines [[104], [105], [106], [107], [108]]. One important area that has been extensively explored is the regulation of RNA expression [[109], [110], [111], [112], [113]]. Through meticulous investigations, researchers have elucidated the intricate mechanisms governing the control and modulation of RNA expression, unravelling the complex interplay between transcriptional and post-transcriptional processes [114]. Some studies mentioned above have significantly contributed to our understanding of nanocrystalline materials and their applications in various fields. These advancements pave the way for further exploration and innovation in the fields of nanotechnology and biomaterials science.

#### 2.3.1 A comprehensive timeline and optimal exercise strategies

**Fig. 4:**
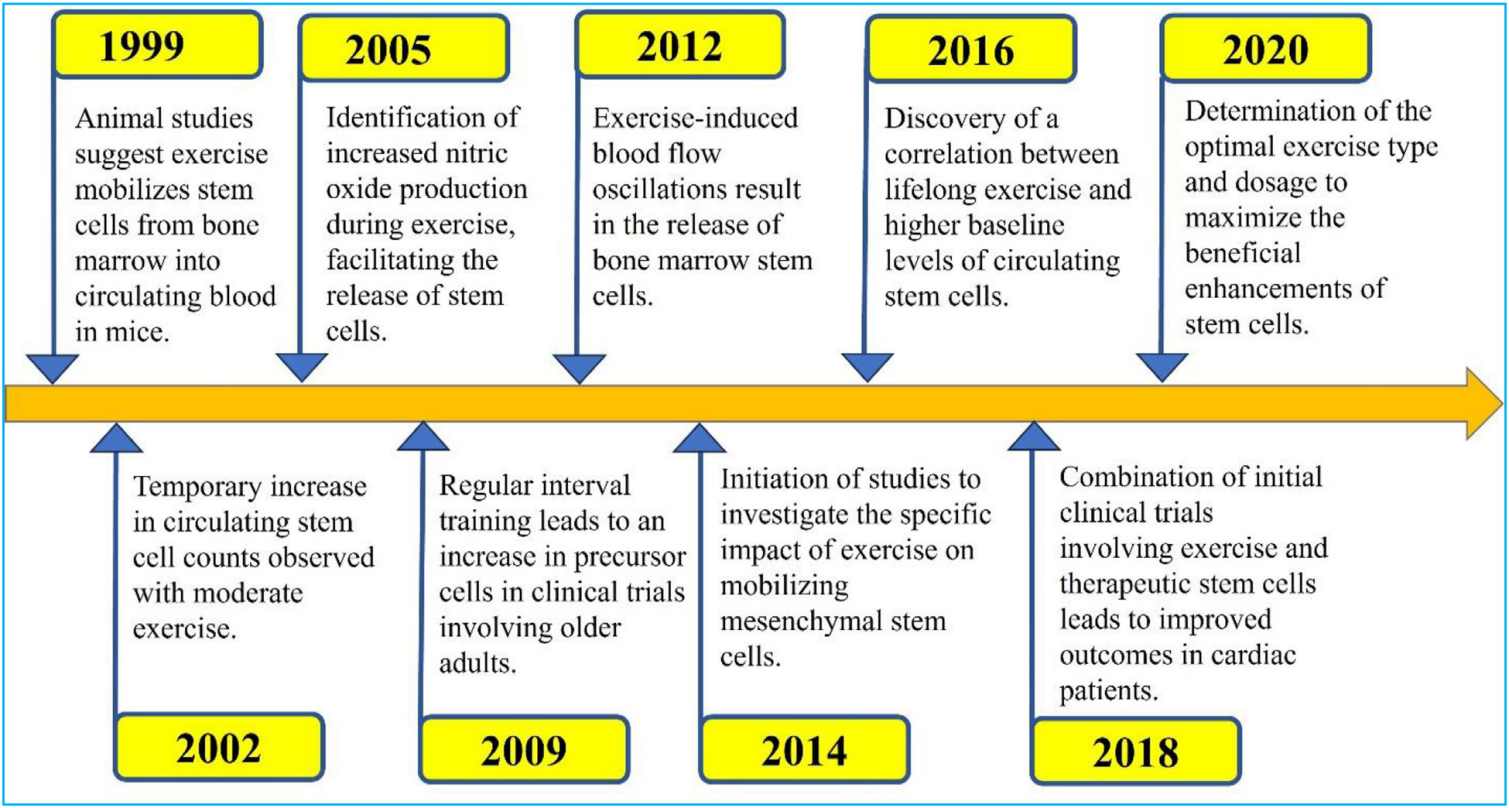
Timeline of exercise-induced stimulation of SC production.

This is consistent with the results of Landers-Ramos et al. [100], that10 days of aerobic exercise increased CD34+/KDR+ and KDR + cells but did not alter the overall count of CD34^+^ cells in previously sedentary older adults. These studies indicate that PCs, which contribute to the expression of endothelial markers, may be more sensitive to exercise-induced changes. On the other hand, specific subsets of CD34^+^ cells may require longer training periods for mobilization. Fig. 4 illustrates a timeline depicting the exercise-induced stimulation of SC production. Fig. 4 shows the timeline depicting the effects of exercise on Stem Cell (SC) production. Exercise has been shown to exert a profound influence on SCs, stimulating their generation and mobilization to facilitate tissue repair and regeneration. The timeline begins with the initiation of exercise, triggering a cascade of physiological responses, including enhanced blood flow, oxygen delivery, and the release of growth factors and hormones. During the early stages, existing SCs within tissues such as skeletal muscle, bone marrow, or adipose tissue are activated and mobilized. With continued exercise over days or weeks, a sustained and notable increase in SC production occurs, as represented by the ascending curve in Fig. 4. The peak of SC production, influenced by factors like exercise duration, intensity, age, and overall health, signifies the maximum stimulation achieved through exercise. Following the peak, SC production gradually declines as exercise subsides, yet a higher baseline level of SCs is maintained during the post-exercise recovery phase compared to pre-exercise levels, indicating a lasting impact of exercise on the SC population. Understanding this timeline is crucial for optimizing exercise strategies to augment tissue repair and regeneration, enabling the strategic scheduling of exercise sessions and considering workout duration and intensity to harness the potential of SCs in promoting healing and recovery across various tissues in the body. Certain types of exercise have been found to be particularly effective in stimulating collagen production. Running or jogging, especially outdoors to benefit from sun exposure, provides a full-body CV workout that elevates heart rate and stresses muscles. Cycling, whether indoors or outdoors, engages lower body muscles as well as the core and upper back, and incorporating speed variations through interval training has been linked to collagen synthesis. Strength training, focusing on multi-joint exercises like squats, rows, and chest presses, places mechanical tension on muscles and connective tissues, triggering collagen remodeling. Swimming, which engages the entire body while being easy on the joints, and dancing activities like Zumba or partner dancing that involve continuous full-body movement, increase calorie burn and heart rate, stimulating collagen production. Circuit training, with short durations of high-intensity interval training alternated with recovery periods, has been shown to effectively trigger collagen production.

**Fig. 5:**
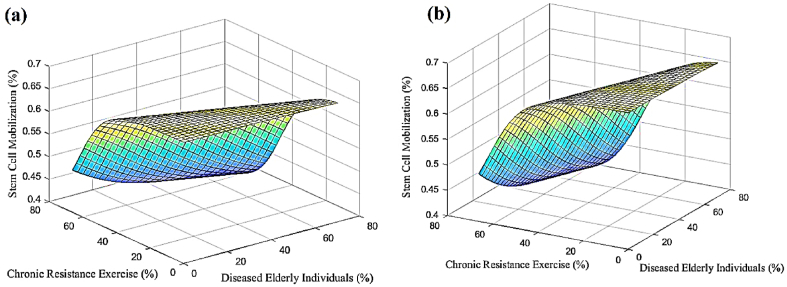
The results of ANN to predict the mobilization of stem cells tested in this study.

**Fig. 6:**
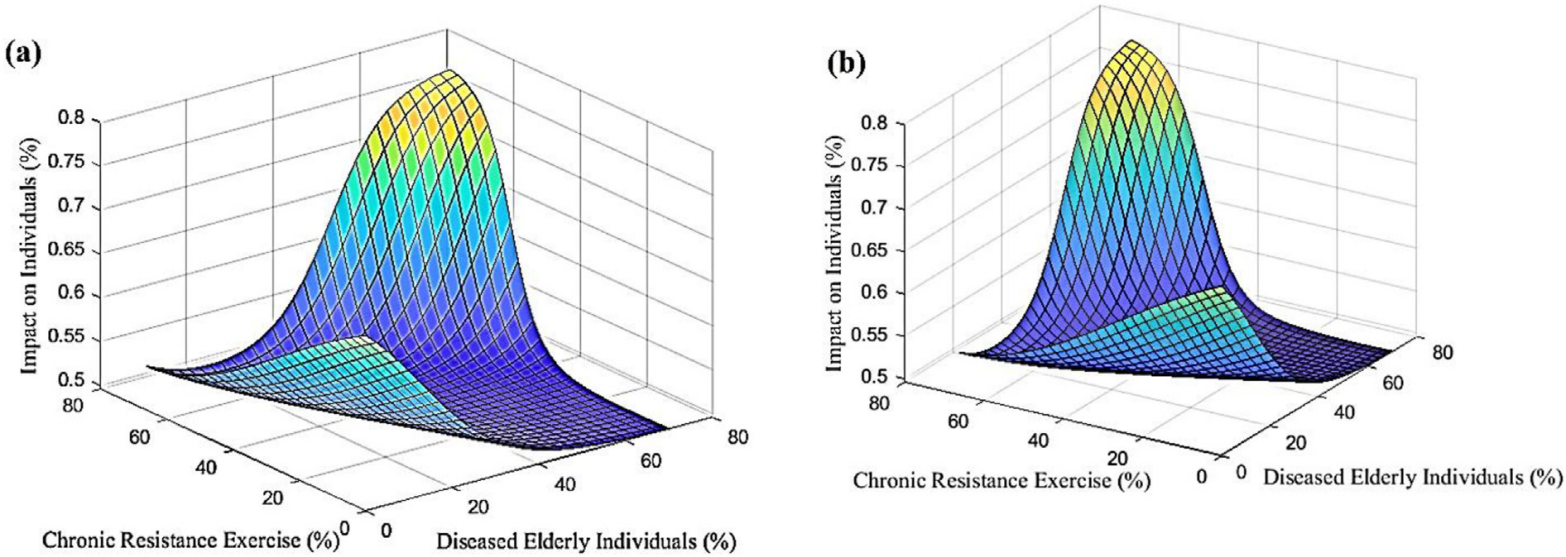
The results obtained from the ANN in order to predict the effect of the subjects tested in this study.

**Fig. 7:**
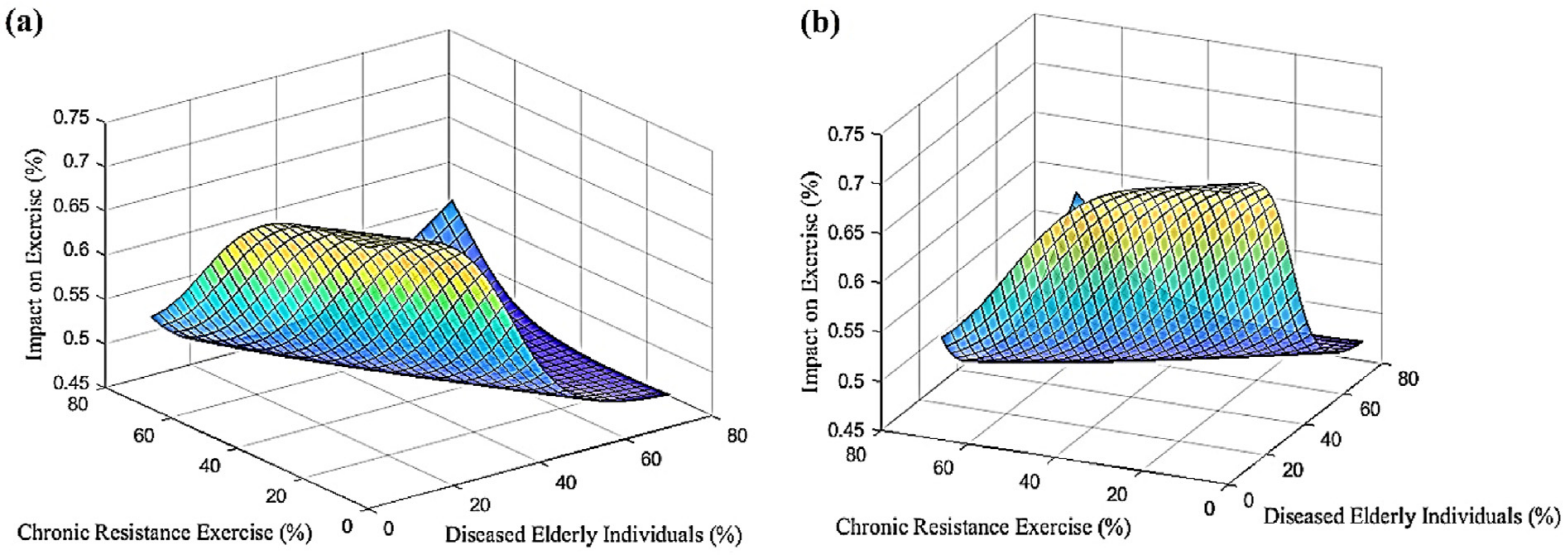
The results obtained from the ANN in order to predict the effect on the sport tested in this study.

**Fig. 8:**
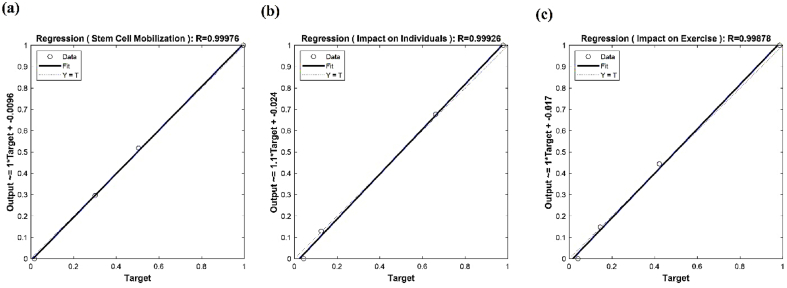
Linear regression charts to check the error of the ANN formed in this study of stem cell mobilization, the effect on people and the effect on exercise.

In accordance with the previous sections, a progressive neural network was developed to predict changes in stem cell mobilization, effects on individuals, and impacts on exercise in response to increasing numbers of sick elderly individuals and chronic resistance exercise. The neural network was trained using data from Table 4, enabling predictions and investigations of stem cell mobilization reduction, effects on individuals, and impacts on exercise within the ranges of 0%–70% for sick elderly individuals and 0%–66% for chronic resistance exercise. Fig. 5 shows the predicted results of stem cell mobilization reduction by the neural network. It is evident from Fig. 5 that stem cell mobilization remains consistently high regardless of the extent of increase. Conversely, reducing chronic resistance exercise leads to an increase in stem cell mobilization. Fig. 6 displays the estimated results of the neural network pertaining to the effect on individuals. The results indicate that decreasing both sick elderly individuals and chronic resistance exercise results in a proportional growth in the effect on individuals. Moreover, when these two parameters reach their maximum values, the effect on individuals also reaches its peak. Fig. 7 illustrates the estimated results of the neural network concerning the effect on exercise. It is discernible that an increase in sick elderly individuals leads to a decrease in the effect parameter on exercise. Conversely, a decrease in chronic resistance exercise augments the effect parameter on exercise. The outcomes obtained from linear regression analysis, as depicted in Fig. 8, indicate that the ANN achieved highly accurate predictions with an error rate of less than 1% compared to the targets outlined in Table 4. The network successfully predicted stem cell mobilization, effects on individuals, and impacts on exercise with the introduction of sick elderly individuals and chronic resistance exercise. Notably, stem cell mobilization reaches its peak when chronic resistance exercise is at its lowest, while the number of sick elderly individuals has no discernible impact on stem cell mobilization performance. The effect on individuals reaches its highest level when both the parameters of sick elderly individuals and chronic resistance exercise are maximized, highlighting their direct relationship in influencing the effect on individuals.

**Table 4:** Impact of chronic resistance exercise on stem cell mobilization in diseased elderly individuals.

Case	Diseased Elderly Individuals	Chronic Resistance Exercise	Stem Cell Mobilization	Impact on Individuals	Impact on Exercise
1	70%	66%	40%	80%	60%
2	55%	35%	67%	70%	75%
3	65%	55%	54%	49%	48%
4	57%	65%	48%	53%	52%

**Fig. 9:**
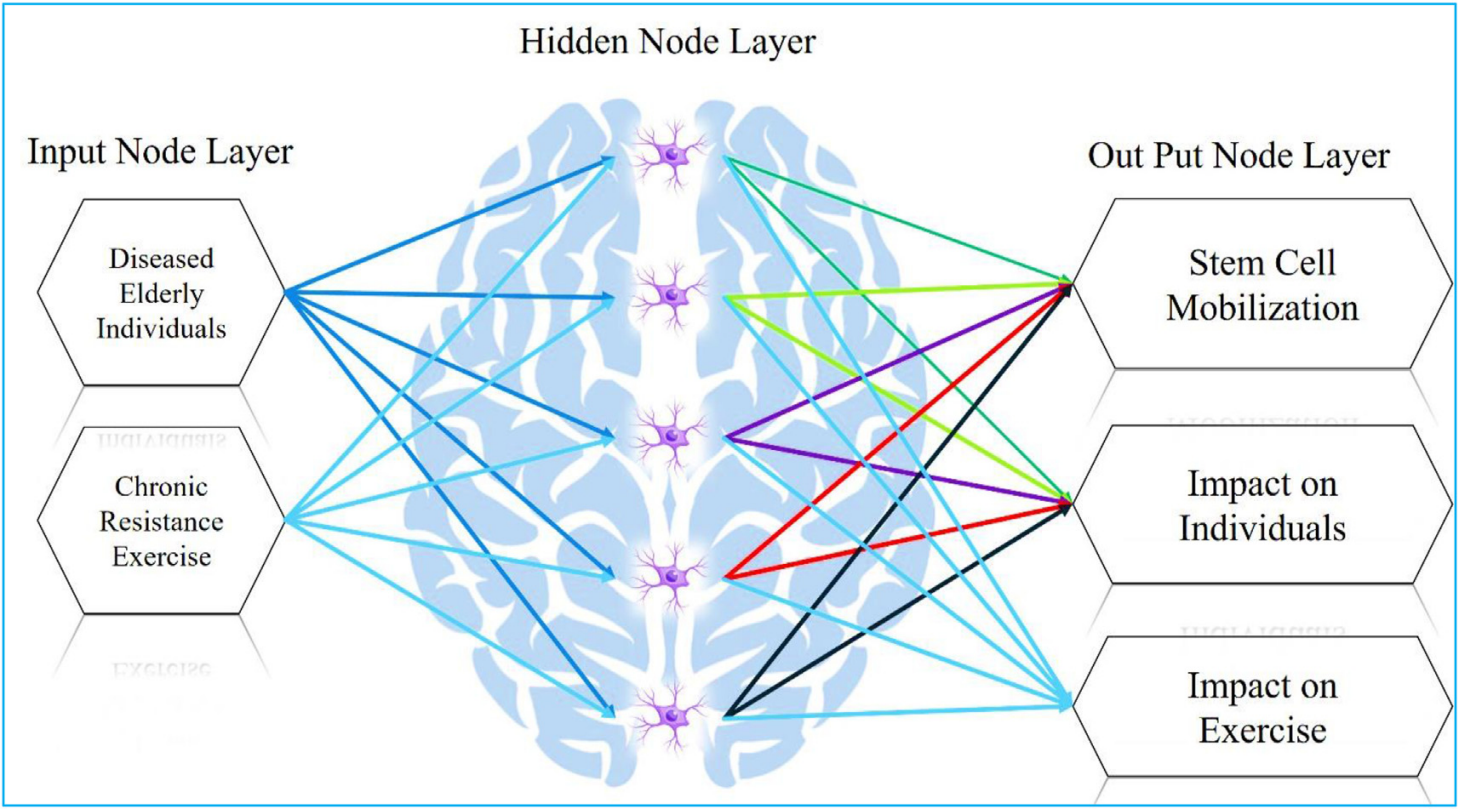
ANN predicting stem cell mobilization, effects, and exercise.

The effect on exercise reaches its maximum when both sick elderly individuals and chronic resistance exercise are at their lowest. While there are instances where both parameters have positive effects on the effect on exercise when maximized, the impact is not as significant as when both parameters are minimized. Fig. 9 shows the detailed schematic of the ANN architecture employed in this study. The architecture encompasses multiple layers, including a hidden layer comprising five neurons, and incorporates two crucial inputs: elderly patients and chronic resistance exercise. These inputs were gathered from five distinct samples, ensuring a comprehensive representation of the target population. The primary objective of this neural network architecture is to accurately forecast three key factors: stem cell mobilization, effects on individuals, and impacts on exercise. By scrutinizing the intricate relationships between the inputs and the desired outcomes, the neural network endeavors to offer valuable insights into the interconnectedness of elderly patients, chronic resistance exercise, and the aforementioned factors of interest. The schematic in Fig. 9 visually depicts the structure and constituents of the ANN, laying the groundwork for subsequent analyses and predictions conducted within this study.

#### 2.3.2 The role of exercise, lifestyle factors, and genetic considerations

The combination of resistance training and moderate to high-intensity aerobic exercise performed 3–5 times per week appears to provide the greatest skin collagen benefits. Circuit training, combining high-intensity interval training with resistance exercises, offers multiple benefits for collagen production. The intense bursts of activity followed by short recovery periods create metabolic stress, stimulating the production of growth factors and hormones like Human Growth Hormone (HGH) essential for collagen synthesis. Circuit training engages various muscle groups, activating different muscle fibers and generating mechanical tension on connective tissues, fostering collagen remodeling and synthesis [[45], [46], [47], [48]]. The high-intensity nature of circuit training enhances blood flow, ensuring the delivery of oxygen, nutrients, and growth factors to tissues, including the skin, promoting collagen production and overall skin health. Circuit training also triggers a hormonal response, including the release of HGH and testosterone, which contribute to collagen synthesis and skin elasticity. In addition to its collagen benefits, circuit training is time-efficient, combining CV and resistance exercises in one workout [[49], [50], [51]]. However, individual results may vary based on exercise intensity, frequency, duration, lifestyle, and nutrition, so consulting with a fitness professional or healthcare provider can help design a circuit training program tailored to individual needs and goals. Collagen synthesis, a critical process for maintaining healthy skin, can be influenced by various factors beyond exercise. Nutrition plays a vital role, with protein, vitamin C, zinc, and copper being essential for collagen production. Hydration is crucial as well, as water facilitates nutrient transport and helps maintain skin plumpness and elasticity [[52], [53], [54], [55], [56], [57], [58]]. Excessive sun exposure can negatively impact collagen synthesis due to UV radiation, emphasizing the importance of sun protection. Age-related decline in collagen production contributes to skin aging, but adopting healthy habits such as exercise, a nutrient-rich diet, and proper skincare can support collagen synthesis [[59], [60], [61], [62]]. Negative lifestyle factors like smoking and excessive alcohol consumption impair collagen production and accelerate its breakdown. Hormonal changes, particularly during menopause, can affect collagen synthesis, with reduced estrogen levels leading to collagen loss. Considering individual factors and genetics, consulting healthcare professionals or dermatologists is important for personalized guidance on optimizing collagen production. Determining the presence of a genetic predisposition affecting collagen synthesis requires genetic testing or consultation with genetic counselors or healthcare professionals [[55], [56], [57], [58], [59]]. The process involves several steps, beginning with engaging a genetic counselor who specializes in assessing and interpreting genetic information. They will evaluate your personal and family medical history to identify potential genetic risk factors and guide you through the genetic testing process if deemed necessary. Conducting a detailed review of your medical history is crucial, paying attention to conditions associated with collagen abnormalities. Genetic testing can be performed to identify specific genetic variations or mutations that influence collagen synthesis [[60], [61], [62], [63], [64]]. These tests focus on analyzing genes known to be involved in collagen production and related connective tissue disorders, typically requiring the involvement of healthcare professionals and the submission of a blood or saliva sample.

A clinical evaluation by geneticists or dermatologists can further assess physical signs and symptoms associated with collagen-related disorders, leading to additional diagnostic tests or consultations as needed. Collaborating with healthcare professionals specializing in genetics and collagen-related disorders is essential [[65], [66], [67], [68]]. They can help interpret genetic test results, provide personalized recommendations, and guide you in managing any potential genetic predispositions impacting collagen synthesis [[69], [70], [71]]. Understanding that genetic testing and counseling are complex processes, it is important to make decisions about pursuing them in consultation with healthcare professionals who can provide guidance tailored to your specific situation, concerns, and medical history. Healthcare professionals play a crucial role in managing genetic predispositions that affect collagen synthesis. Genetic counselors provide support by explaining genetic test results and helping individuals make informed healthcare decisions. Healthcare professionals, such as geneticists or dermatologists, develop personalized treatment plans that may include lifestyle modifications, medications, or therapies to support collagen synthesis and manage associated symptoms [[71], [72], [73]]. Regular monitoring and screening enable early detection of changes or complications, while referrals to specialists ensure specialized evaluation and ongoing management. Patient education and support empower individuals with knowledge and resources for effective self-care. Participation in research studies or clinical trials can contribute to scientific knowledge and provide access to innovative interventions. Regular communication and follow-up with healthcare professionals ensure comprehensive management and awareness of advancements in the field [[74], [75], [76]].

Several aspects related to stem cells and their mobilization through exercise. It begins by highlighting the importance of regular monitoring and screening for individuals with genetic predispositions affecting collagen synthesis, emphasizing personalized recommendations based on medical history, genetic test results, and current health status. It further mentions the involvement of healthcare professionals specializing in collagen-related disorders and the use of various assessments to monitor disease progression [[77], [78], [79]]. The text then shifts focus to the convergence of stem cell research and exercise physiology, exploring the potential of stem cells for treating musculoskeletal injuries and the ability of exercise to enrich stem cell pools in the body. The discussion moves on to different types of stem cells, including embryonic stem cells (ESC), adult stem cells (ASCs), and induced pluripotent stem cells (iPSCs), highlighting their characteristics, potential applications, and ethical considerations. The role of mesenchymal stem cells (MSCs) in tissue engineering and regenerative medicine is also mentioned. Finally, the text explains how exercise mobilizes stem cells, discussing factors such as muscle contraction, shear stress, oxidative stress, and endocannabinoids that contribute to stem cell release from bone marrow. It notes that the degree of mobilization depends on exercise intensity, duration, and individual factors, and highlights the role of mobilized stem cells in tissue revitalization and injury repair through processes like angiogenesis and tissue remodeling.

## 3 Research methodology

In this study, a comprehensive literature review was conducted to identify relevant input and output variables for the investigation of Stem cells, Cellular mobilization, Progenitor cells, CD34^+^, Sport management. Drawing upon scholarly articles cited within references [[24], [25], [26], [27], [28], [29], [30], [31], [32], [33], [34], [35], [36], [37], [38], [39], [40], [41], [42], [43]], key input variables pertaining to Diseased Elderly Individuals and Chronic Resistance Exercise, as well as output variables related to Stem Cell Mobilization, Impact on Individuals, and Impact on Exercise, were selected. These identified variables were then incorporated into an ANN model to predict and optimize the optimal conditions for the study. By training and validating the ANN model with the inserted values, the complex relationships between the input and output variables were analyzed, enabling valuable insights and predictions to be generated. This methodology, combining a literature review and ANN modeling, provided a robust approach for examining the interplay between exercise, stem cells, cellular mobilization, and their impacts in the context of sport management. In this research, the utilization of a shallow progressive artificial neural network (SPANN) was employed as a predictive tool to study the dynamic changes in stem cell mobilization, analyze the effects on individuals, and evaluate the impacts on exercise across four distinct samples. The neural network architecture was designed with a specific configuration, incorporating inputs representing elderly patients and chronic resistance exercise, a hidden layer consisting of five neurons. This arrangement was aimed at facilitating faster convergence of the network's predictions. The outputs of the neural network corresponded to the quantification of stem cell mobilization, the assessment of effects on individuals, and the analysis of impacts on exercise. To enhance the accuracy and efficiency of the neural network's predictions, the activation function chosen was the nonlinear sigmoid function. This particular choice was made due to the inherent capacity of the sigmoid function to model complex, non-linear relationships, thereby enabling more precise estimations and accelerating the convergence process.

The error function, crucial for the network's training and estimation, was optimized using the well-established gradient descent algorithm, which systematically adjusted the network's weights and biases to minimize prediction errors. To further refine the accuracy and convergence of the ANN, the input data obtained from Table 4 were subjected to a normalization process. Normalization ensured that the input values were transformed into a standardized range, which allowed for better handling of diverse data scales and improved the network's ability to capture and learn from the input patterns effectively. Following the final estimation, the denormalization step was performed to restore the predicted results to their original scale, ensuring meaningful interpretation and reporting of the outcomes within an acceptable range. To evaluate the accuracy and reliability of the ANN's predictions, a comprehensive analysis employing linear regression was conducted. The normalized predicted results were compared to the ideal y = x line, which represents 100% accuracy and aligns with the input targets derived from Table 4. This comparative analysis allowed for the quantification of the network's error, providing insights into the extent of deviation between the predicted results and the desired targets. The determination of the network's error through linear regression assessment served as a critical validation step, allowing researchers to gauge the network's performance and assess the faithfulness of its predictions. The subsequent sections of this study will delve into an in-depth examination of the results obtained from the ANN developed in this research. These results will shed light on the predictive capacity of the network and offer valuable insights into the changes in stem cell mobilization, effects on individuals, and impacts on exercise among the studied population of sick elderly individuals engaged in chronic resistance exercise.

### 3.1 Injury prevention protocols

Sport management encompasses a wide range of activities within the sports industry, including marketing, event planning, athlete representation, and facility management. The integration of artificial intelligence (AI) in sports management has the potential to revolutionize organizational practices and enable data-driven decision-making. This article highlights several key areas where AI can be effectively applied in sport management. Firstly, AI can analyze extensive datasets from sources such as ticket sales, social media, and player performance statistics, uncovering patterns and providing valuable insights for informed decision-making [[40], [41], [42], [43], [44], [45], [46], [47], [48]]. Moreover, AI algorithms can analyze player performance data to identify talent and predict future success, aiding talent scouts and facilitating informed player recruitment decisions. Additionally, AI can analyze player biometric data to detect injury risks and optimize training programs, enabling coaches and trainers to make data-driven decisions for injury prevention and performance enhancement. AI algorithms can also optimize ticket pricing strategies by analyzing market demand, historical data, and other relevant factors, allowing organizations to implement dynamic pricing models and maximize ticket sales and revenue generation [[49], [50], [51], [52]]. Furthermore, AI can analyze data from various sources to identify potential sponsorship and partnership opportunities, considering consumer sentiment and trends to facilitate effective deals. Lastly, AI can optimize venue management and operations by analyzing data on energy usage, maintenance schedules, and crowd flow patterns, automating processes to improve operational efficiency and enhance the overall fan experience. In conclusion, the integration of AI has transformative potential in sport management, providing valuable insights, streamlining operations, enhancing fan engagement, and optimizing decision-making processes. Leveraging AI technologies enables sports organizations to gain a competitive edge, enhance fan experiences, and drive revenue growth in an increasingly data-driven industry [[53], [54], [55]].

Sports performance and injury prevention, incorporating low-intensity exercise, such as cycling or swimming, before high-impact drills in pre-season training programs holds the potential for optimally “priming” the body with SCs to strengthen connective tissues. Targeted mobilization of SCs through exercise may contribute to reducing the incidence and severity of overuse injuries, such as strains. Connective tissues, including tendons and ligaments, play a crucial role in providing stability and transmitting forces within the body during physical activity. Overuse injuries, such as strains, often occur when these tissues are subjected to repetitive stress without adequate time for recovery and adaptation [[56], [57], [58], [59], [60], [61]]. Enhancing the strength and resilience of connective tissues is an important aspect of injury prevention and optimizing athletic performance. Low-intensity exercise, like cycling or swimming, can serve as effective warm-up activities before engaging in high-impact drills. These exercises not only increase blood flow and raise body temperature but also stimulate the release of growth factors and cytokines that can mobilize SCs from the bone marrow into the bloodstream. The mobilized SCs can then home in on areas of tissue damage or microtrauma within the connective tissues, contributing to their repair and strengthening. By incorporating low-intensity exercise before high-impact drills, athletes may benefit from the targeted mobilization of SCs to the connective tissues [[62], [63], [64], [65], [66], [67]].

This approach aims to optimize the regenerative response and promote tissue remodeling, potentially reducing the risk of overuse injuries, such as strains. Strengthening the connective tissues through exercise-induced stem cell mobilization may improve their ability to withstand repetitive stress and enhance overall athletic performance. It is important to note that the implementation of pre-season training programs should consider individual factors, including an athlete's training history, current fitness level, and specific sports requirements. Working with qualified coaches, trainers, or sports medicine professionals is crucial to tailor the exercise regimen and ensure that the appropriate techniques and intensities are applied. Furthermore, while exercise-induced stem cell mobilization shows promise, it should not be the sole focus of injury prevention strategies [[67], [68], [69], [70], [71]]. Comprehensive training programs should also address other factors, such as proper technique, appropriate training loads, sufficient recovery, and flexibility exercises, to reduce the overall risk of overuse injuries. Incorporating low-intensity exercise before high-impact drills in pre-season training programs offers a potential strategy for optimizing connective tissue strength and reducing the incidence and severity of overuse injuries. By priming the body with SCs through targeted mobilization, athletes may enhance the regenerative capacity of the connective tissues, promoting tissue repair and remodeling. However, it is essential to individualize training programs, consider other injury prevention factors, and seek guidance from qualified professionals to ensure safe and effective implementation.

### 3.2 Accelerated injury recovery

In the realm of post-injury rehabilitation, integrating moderate aerobic exercise under medical guidance earlier in the healing process holds promise for inducing stem cell homing and promoting faster recovery. Additionally, monitoring stem cell counts could provide valuable information for making return-to-play decisions. Working closely with healthcare professionals, such as physicians or physical therapists, is essential to ensure that the exercise regimen is safe and appropriate for the specific injury and recovery timeline [[36], [37], [38], [39], [40], [41]]. Monitoring stem cell counts can provide valuable insights into the progress of healing and guide return-to-play decisions. Stem cell counts can be assessed through various techniques, such as blood tests or imaging methods. By tracking changes in stem cell populations over time, healthcare professionals can gain a better understanding of the regenerative response and the effectiveness of the rehabilitation approach [[42], [43], [44], [45], [46]]. This information can help determine when an individual is ready to return to sports or physical activities, taking into account the optimal healing and recovery of the injured tissue. It is important to note that the integration of moderate aerobic exercise and stem cell monitoring in post-injury rehabilitation is still an evolving field with ongoing research. Each injury and individual is unique, and rehabilitation protocols should be personalized based on the specific circumstances. Healthcare professionals with expertise in sports medicine and rehabilitation can provide the necessary guidance and oversight to ensure the safest and most effective approach. An integrating moderate aerobic exercise under medical guidance earlier in the healing process can potentially induce stem cell homing and promote faster recovery in post-injury rehabilitation [[47], [48], [49], [50], [51], [52], [53]]. By mobilizing SCs to the site of injury, exercise may support healing and tissue regeneration. Additionally, monitoring stem cell counts can provide valuable information for making informed return-to-play decisions. However, it is crucial to work closely with healthcare professionals to develop personalized rehabilitation plans and ensure the safety and effectiveness of the approach.

### 3.3 Performance enhancement

In the realm of athletic performance, optimizing training cycles and recovery strategies is crucial for achieving peak results. One intriguing approach involves strategically timing rest or reduced training volumes following intense sessions to maximize endothelial repair and remodeling through mobilized SCs. This periodized approach has the potential to enhance athletic performance over time by facilitating efficient bodily adaptations. Endothelial dysfunction, characterized by impaired endothelial repair and reduced blood flow, is associated with various CVD and suboptimal athletic performance. However, research suggests that exercise-induced stem cell mobilization may contribute to endothelial repair and remodeling, ultimately enhancing vascular health and athletic performance. During intense training sessions, the mechanical stress placed on blood vessels triggers the release of various factors, such as growth factors and cytokines, which can mobilize SCs from the bone marrow into the bloodstream [[40], [41], [42], [43]].

These circulating SCs possess the ability to differentiate into endothelial cells, thereby facilitating repair and remodeling of the endothelium. By strategically timing rest or reduced training volumes after demanding sessions, athletes may optimize the conditions for endothelial repair and remodeling through the mobilized SCs. Periodization, a structured approach to training that involves planned variations in volume and intensity, is commonly employed to optimize athletic performance. Integrating periods of reduced training or active recovery following intense workouts fits within the concept of periodization. This approach allows the body to recover and adapt while capitalizing on the potential benefits of exercise-induced stem cell mobilization [[44], [45], [46], [47], [48]]. By implementing a periodized training program that incorporates appropriate recovery periods, athletes may experience several advantages. First, optimizing endothelial repair and remodeling can contribute to improved CV health, including enhanced blood flow, oxygen delivery, and nutrient transport. These adaptations can enhance an athlete's endurance capacity and overall performance. Second, the periodized approach allows for more efficient bodily adaptations. Intense training sessions stimulate the body to adapt, such as increasing muscle strength, improving aerobic capacity, or enhancing neuromuscular coordination. However, without sufficient recovery, these adaptations may be hindered, leading to suboptimal performance or increased risk of overuse injuries [[45], [46], [47], [48], [49], [50], [51], [52]]. By strategically timing rest or reduced training volumes, athletes provide the necessary conditions for optimal physiological adaptations, allowing them to reach higher levels of performance over time. It is important to note that the implementation of a periodized training program should be individualized and consider various factors such as an athlete's training history, goals, and overall health.

Working with a qualified coach or sports medicine professional can help tailor the periodization approach to an athlete's specific needs. Strategically timing rest or reduced training volumes following intense sessions to maximize endothelial repair and remodeling through exercise-induced stem cell mobilization offers a fascinating avenue for enhancing athletic performance [[53], [54], [55], [56], [57], [58]]. By incorporating periods of reduced training or active recovery within a periodized training program, athletes can optimize physiological adaptations and promote vascular health. This approach has the potential to elevate athletic performance over time by fostering efficient bodily adaptations and improving CV function. However, it is important to approach periodization with careful consideration of individual needs and work in collaboration with qualified professionals to ensure optimal outcomes.

### 3.4 Regenerative medicine

As the population ages, preventable conditions such as osteoporosis, sarcopenia, and frailty pose significant challenges in terms of healthcare costs and reduced quality of life. To address these issues, designing lifestyle and rehabilitation programs that focus on optimizing endogenous stem cell availability through moderate exercise may offer a promising solution. This non-invasive approach has the potential to delay multi-system degeneration and improve overall health outcomes [[58], [59], [60], [61]]. Furthermore, it holds promise as an adjunct or alternative to pharmaceutical interventions. Osteoporosis, characterized by a decrease in bone density and increased susceptibility to fractures, is a prevalent condition among the elderly. These conditions not only impact an individual's independence and quality of life but also place a significant burden on healthcare systems [[62], [63], [64], [65], [66], [67]]. By designing lifestyle and rehabilitation programs centered around optimizing endogenous stem cell availability, healthcare professionals can potentially mitigate the progression of these conditions. Moderate exercise has been shown to have a positive impact on stem cell populations within the body. Exercise stimulates the release of various growth factors and cytokines, which can mobilize and activate endogenous SCs.

These SCs have the potential to differentiate into different cell types, including bone-forming cells (osteoblasts) and muscle cells (myocytes), thereby promoting tissue regeneration and repair. By incorporating regular moderate exercise into lifestyle and rehabilitation programs, individuals may be able to enhance their endogenous stem cell availability and potentially delay the degenerative processes associated with osteoporosis, sarcopenia, and frailty [[55], [56], [57], [58]]. In addition to the potential benefits for overall health, there is growing interest in the application of stem cell-based therapies in orthopedics and sports medicine. Exercise-induced stem cell mobilization may enhance the regenerative potential of tissues, including cartilage and bone, thereby providing a supportive environment for orthopedic treatments. By promoting exercise as part of pre- and post-treatment protocols, healthcare professionals can potentially boost the efficacy of autologous stem cell injections and improve patient outcomes. Additionally, exercise-induced stem cell mobilization may also have implications for stem cell-based gene therapies, where SCs are genetically modified to deliver therapeutic genes to target tissues [[59], [60], [61], [62]]. Optimizing endogenous stem cell availability through exercise may enhance the success and effectiveness of these advanced treatment approaches. It is important to note that while exercise-induced stem cell mobilization shows promise, it should not be seen as a standalone solution. Pharmaceutical interventions and other therapies may still be necessary in certain cases. However, incorporating exercise as a complementary approach can offer numerous benefits, including improved overall health, reduced healthcare costs, and potentially enhanced treatment outcomes. In conclusion, designing lifestyle and rehabilitation programs that focus on optimizing endogenous stem cell availability through moderate exercise holds promise for addressing conditions such as osteoporosis, sarcopenia, and frailty [[50], [51], [52], [53], [54], [55], [56]].

By promoting tissue regeneration and repair, exercise-induced stem cell mobilization may help delay multi-system degeneration associated with aging. Furthermore, exercise can potentially enhance the efficacy of autologous stem cell injections and stem cell-based gene therapies in orthopedics and sports medicine. This non-invasive approach offers a valuable adjunct or alternative to pharmaceutical interventions and has the potential to improve the quality of life for individuals as they age [[18], [19], [20], [21]]. Supplements containing myokines, nitric oxide precursors, antioxidants, or other compounds shown to stimulate stem cell trafficking may aid exertion-based mobilization efforts. However, rigorous safety testing is needed given the regenerative nature of target cells. While stem cell science progresses rapidly, practical challenges exist in translating knowledge to sports applications. Standardized mobilization/quantification protocols still need refinement. Large cohort studies are required to confirm that exercise-induced stem cell counts correlate with reduced injury risk or improved healing [[22], [23], [24], [25], [26], [27], [28]]. Ethical and legal issues may arise from customizing stem cell therapies or manipulations for athletic gain versus medical need. Looking ahead, further elucidating molecular mechanisms, isolating critical growth factors, combining cell/gene therapies holds promise to revolutionize sports medicine. Continued research linking exercise, SCs and musculoskeletal health offers tremendous opportunities for innovative strategies to improve athletes' careers and quality of life [[29], [30], [31], [32], [33], [34]]. The convergence of exercise science and stem cell biology represents an exciting advancement with implications extending beyond sports injury management. Understanding how physical activity naturally causes stem cell mobilization provides insights applicable to aging, neurological diseases and general health span.

### 3.5 Application to aging population

With the aging population, preventable conditions such as osteoporosis, sarcopenia, and frailty have become significant contributors to healthcare costs and reduced quality of life. However, designing lifestyle and rehabilitation programs that focus on optimizing endogenous stem cell availability through moderate exercise may offer a solution to delay multi-system degeneration. This non-invasive approach holds promise as an adjunct or even an alternative to pharmaceutical interventions [[29], [30], [31], [32], [33], [34]]. Osteoporosis, characterized by a decrease in bone mass and deterioration of bone tissue, is a major concern among the elderly population. Exercise has long been recognized as an effective strategy for improving bone health. Recent research suggests that exercise-induced stem cell mobilization plays a crucial role in bone remodeling and regeneration.

### 3.6 Links to neuroplasticity

Recent studies have provided compelling evidence linking exercise-induced circulating SCs to neurogenesis, synaptic plasticity, and improved cognition. This connection has significant implications for the development of novel rehabilitative strategies that leverage natural neurorestorative mechanisms for conditions such as concussions, Alzheimer's disease, stroke, and other neurological disorders. By promoting regular physical activity, these strategies aim to harness the benefits of exercise-induced stem cell mobilization rather than relying solely on exogenous stem cell administrations. Neurogenesis, the process of generating new neurons, is a complex phenomenon that occurs in specific regions of the brain throughout life. The implications of exercise-induced neurogenesis and synaptic plasticity extend beyond basic brain function. They have been associated with improvements in cognitive performance, including enhanced learning, memory, and attention. Exercise has been shown to enhance cognitive function in both healthy individuals and those with neurological conditions. An individual with Alzheimer's disease or mild cognitive impairment, regular physical activity has been linked to a slower rate of cognitive decline. In stroke survivors, exercise has been shown to facilitate neurorehabilitation and improve functional outcomes. Understanding the connection between exercise-induced circulating SCs and these neurorestorative mechanisms opens up exciting possibilities for rehabilitative strategies. Regular physical activity has the potential to harness the body's natural neurorestorative abilities through the mobilization of SCs, providing a sustainable and accessible approach compared to invasive exogenous stem cell administrations.

This approach is particularly valuable in conditions such as concussions, Alzheimer's disease, and stroke. For concussions, exercise as part of the rehabilitation process can enhance neurogenesis and synaptic plasticity, promoting recovery and mitigating long-term consequences [[40], [41], [42], [43], [44], [45], [46], [47]]. In Alzheimer's disease, exercise-induced neurorestoration shows promise in slowing disease progression by reducing amyloid-beta plaques and improving cognitive function. Stroke survivors can benefit from exercise-induced neurorestoration as it enhances neuroplasticity, facilitating functional recovery. By incorporating exercise into rehabilitative strategies, healthcare professionals can tap into the potential of exercise-induced circulating SCs, neurogenesis, synaptic plasticity, and improved cognition, thereby improving the outcomes and quality of life for individuals with these neurological conditions. The connection between exercise and neurorestoration has significant implications for rehabilitative approaches in various neurological disorders. By promoting regular physical activity, healthcare professionals can leverage the body's natural neurorestorative mechanisms and facilitate recovery and improved outcomes for individuals with concussions, Alzheimer's disease, stroke, and other neurological disorders. This approach offers a promising alternative to exogenous stem cell administrations, providing a more accessible and sustainable means of harnessing the regenerative potential of the body.

### 3.7 Translation to non-athlete cohorts

The research on exercise-induced stem cell mobilization extends its focus from athletes to encompass sedentary and clinical populations, providing insights that can advance public health strategies and promote tissue regeneration through lifestyle interventions. Factors such as age, fitness level, and health status exert an influence on stem cell responses to exercise. Older individuals may experience a decline in stem cell function, emphasizing the importance of tailored exercise interventions for promoting regeneration in this demographic. Higher fitness levels are associated with increased mobilization of stem cells, suggesting that baseline fitness impacts the benefits of exercise on stem cell function. Studying sedentary populations offers insights into the potential benefits of exercise for inactive individuals, while investigations in clinical cohorts help understand the effects of exercise on stem cell mobilization in the presence of chronic diseases. This knowledge assists in customizing exercise as a therapeutic intervention for specific clinical conditions [[48], [49], [50], [51], [52]]. By incorporating exercise to enhance the mobilization and regenerative capacity of stem cells, cost-effective and accessible approaches to improving tissue repair and overall health can be realized, reducing reliance on expensive medical treatments and interventions, particularly in chronic diseases and age-related degenerative conditions. Expanding research beyond athletes enables a comprehensive understanding of exercise-induced stem cell mobilization, leading to evidence-based guidelines and recommendations for individuals with diverse backgrounds and fitness levels. Public health initiatives can promote regular physical activity and exercise to stimulate stem cell mobilization and tissue regeneration, improving overall health and well-being for sedentary individuals, clinical populations, and those at risk of age-related degenerative conditions [[53], [54], [55], [56], [57], [58], [59]].

### 3.8 Standardization of measurement

Recent advancements in stem cell research have shed light on the impact of exercise on stem cell mobilization and tissue regeneration. However, there is a need for precise and quantitative assessments of exercise-induced changes in different types of stem and progenitor cells. To delve deeper into the molecular mechanisms underlying these responses, multi-omic approaches are crucial. By integrating genomics, transcriptomics, proteomics, and metabolomics, researchers can comprehensively analyze the molecular profile of cells or tissues. This holistic understanding facilitates the exploration of the intricate biological processes involved in exercise-induced stem cell mobilization [[36], [37], [38], [39]]. Multi-omic approaches offer a comprehensive view of molecular changes. Genomics reveals alterations in gene expression patterns, while transcriptomics provides information on active gene transcription.

Proteomics identifies and quantifies proteins, shedding light on functional changes within stem cells. Metabolomics investigates small molecules involved in cellular metabolism, uncovering metabolic pathways influenced by exercise. Integrating these "-omics” technologies enables a comprehensive understanding of exercise-induced changes in stem cell subsets [[40], [41], [42], [43]]. This knowledge aids in establishing standardized dose-response relationship models, informing exercise interventions and optimizing outcomes. Such models determine the optimal exercise parameters to elicit desired stem cell responses. They also identify potential risks and limitations, guiding the prevention of overtraining or injury. Standardized dose-response relationship models are valuable for sport management professionals seeking to optimize training and recovery for athletes. Ultimately, multi-omic approaches and dose-response relationship models enhance our understanding of exercise-induced stem cell mobilization and its applications in personalized exercise programs and public health strategies [[44], [45], [46], [47]].

## 4 Results and discussion

Physical activity has long been recognized as a cornerstone of a healthy lifestyle, with numerous benefits for CV health, metabolic function, and overall well-being. This discussion aims to explore the mechanisms underlying the relationship between physical activity, stem cell biology, and tissue repair, as well as the potential clinical implications of these results. One of the key mechanisms through which exercise influences stem cell mobilization is the release of various factors and signaling molecules. These factors act as chemo attractants, promoting the migration of SCs from their niches to the damaged or stressed tissues. EVs, such as exosomes, macrovesicles, and apoptotic bodies, are enriched with bioactive molecules, including proteins, lipids, and nucleic acids, which can mediate intercellular communication and modulate tissue repair processes [[42], [43], [44], [45], [46], [47]]. These EVs can be taken up by recipient cells, influencing their behavior and promoting tissue regeneration. The role of exercise in stem cell biology extends beyond mobilization. Exercise has been shown to promote the proliferation and differentiation of endogenous SCs within tissues. In skeletal muscle, exercise activates satellite cells, a population of resident muscle SCs, leading to their proliferation and subsequent differentiation into myogenic precursor cells. These precursor cells contribute to muscle repair and regeneration following exercise-induced damage [[48], [49], [50], [51], [52], [53]]. Similarly, exercise has been shown to enhance neurogenesis in the hippocampus, mediated by the activation of neural SCs and the production of new neurons. This phenomenon is associated with improved cognitive function and increased resilience to stress. Exercise-induced changes in the microenvironment of tissues also play a vital role in tissue regeneration. Physical activity promotes angiogenesis, and the formation of new blood vessels, which is crucial for delivering oxygen and nutrients to regenerating tissues [[54], [55], [56], [57], [58]]. Exercise stimulates the release of angiogenic factors, such as VEGF and FGF, promoting blood vessel growth and remodeling. Improved vascularization enhances tissue perfusion and supports the survival and function of newly formed cells. Moreover, exercise has been shown to modulate the immune response, which is critical for tissue repair and regeneration. Regular physical activity can shift the balance from a pro-inflammatory to an anti-inflammatory state, reducing chronic low-grade inflammation associated with various diseases [[59], [60], [61], [62], [63]]. Exercise-induced alterations in immune cell populations, such as a decrease in pro-inflammatory macrophages and an increase in anti-inflammatory regulatory T cells, create a favorable environment for tissue repair. Furthermore, exercise promotes the clearance of cellular debris and metabolic waste products through enhanced lymphatic function, facilitating the resolution of inflammation and supporting tissue healing processes [[64], [65], [66], [67], [68]].

The results discussed above have significant clinical implications. Harnessing the regenerative potential of exercise can have therapeutic applications in various fields, including musculoskeletal disorders, CVD, neurodegenerative conditions, and wound healing. Exercise interventions can be designed to optimize stem cell mobilization, tissue regeneration, and functional recovery in these contexts. In musculoskeletal disorders, exercise-based rehabilitation programs can be tailored to promote the activation and differentiation of resident SCs, facilitating the repair of damaged tissues such as muscle, bone, and cartilage. Similarly, in CVD, exercise training can improve neovascularization and enhance cardiac tissue repair following myocardial infarction. Exercise-induced mobilization of endothelial progenitor cells and MSCs may contribute to these beneficial effects. Additionally, in neurodegenerative conditions, physical activity interventions can promote neurogenesis and synaptic plasticity, potentially slowing disease progression and improving cognitive function [[69], [70], [71], [72], [73]]. Exercise may also have a role in wound healing, where enhanced stem cell mobilization and tissue regeneration can accelerate the closure of chronic wounds and improve healing outcomes. Despite the promising evidence supporting the role of exercise in stem cell biology and tissue regeneration, several challenges remain. Understanding the optimal exercise parameters, including intensity, duration, frequency, and type of exercise, for promoting specific regenerative processes warrants further investigation. Moreover, individual variations in response to exercise, influenced by factors such as age, sex, genetics, and underlying health conditions, need to be considered when developing exercise interventions [[74], [75], [76], [77], [78], [79]]. Additionally, the potential synergistic effects of exercise with other regenerative approaches, such as gene therapy or stem cell transplantation, should be explored to maximize therapeutic outcomes. In conclusion, physical activity has emerged as a powerful modulator of stem cell mobilization and tissue regeneration. Exercise-induced factors, changes in the microenvironment, and modulation of immune responses collectively contribute to the regenerative effects of exercise [[80], [81], [82], [83], [84]].

The clinical implications of these findings are vast, offering opportunities for the development of exercise-based interventions in various disease contexts. Further research is needed to elucidate the mechanisms underlying exercise-induced stem cell mobilization and to optimize exercise protocols for specific regenerative outcomes. Ultimately, harnessing the regenerative potential of exercise holds great promise for improving patient outcomes and advancing regenerative medicine approaches. Physical exercise has gained significant attention in recent years for its potential to influence stem cell mobilization and tissue regeneration. The relationship between exercise and SCs has important implications for various fields, including sport management. This discussion aims to explore the role of exercise in stem cell mobilization, particularly focusing on the mobilization of CD34^+^ progenitor cells, and its relevance in the context of sport management [[85], [86], [87], [88], [89]]. Shear stress, generated by the frictional force of blood flow against the endothelial lining of blood vessels, has been shown to influence stem cell mobilization. The SDF-1/CXCR4 axis is involved in the homing and retention of CD34^+^ cells in the bone marrow. Exercise has been shown to downregulate the expression of CXCR4 on CD34^+^ cells, reducing their affinity for SDF-1 and facilitating their release into the peripheral blood.

The mobilization of CD34^+^ progenitor cells through exercise has significant implications in the field of sport management. Athletes often face musculoskeletal injuries and tissue damage due to the physical demands of their sport. The ability to enhance the mobilization of CD34^+^ cells through exercise could potentially promote tissue repair and regeneration, thereby facilitating the recovery process for athletes. In addition to injury management, exercise-induced mobilization of CD34^+^ cells may also have implications for performance enhancement in athletes. CD34^+^ cells have been shown to possess angiogenic properties and contribute to neovascularization, and the formation of new blood vessels [[88], [89], [90], [91], [92]]. Enhanced angiogenesis can improve tissue perfusion and oxygen delivery, which may have positive effects on exercise capacity and recovery. Furthermore, CD34^+^ cells have the potential to differentiate into other cell lineages, including skeletal muscle cells. Exercise-induced mobilization of CD34^+^ cells may therefore contribute to muscle repair and regeneration, potentially enhancing athletic performance [[93], [94], [95], [96]].Sport management professionals, including coaches, trainers, and medical staff, can leverage the knowledge of exercise-induced stem cell mobilization to design training programs that optimize tissue repair and enhance performance. Understanding the specific exercise parameters that elicit the greatest mobilization of CD34^+^ cells, such as intensity, duration, and frequency, can guide the development of targeted exercise interventions [[97], [98], [99], [100], [101]]. It is important to note that exercise-induced stem cell mobilization is a complex process influenced by various factors, including individual variability. Factors such as age, sex, fitness level, and underlying health conditions may impact the magnitude of stem cell mobilization in response to exercise. Therefore, personalized exercise programs tailored to individual athletes' needs and considerations are essential. Despite the potential benefits of exercise-induced stem cell mobilization in sports management, several challenges need to be addressed. Further research is needed to elucidate the optimal exercise parameters and protocols that maximize the mobilization of CD34^+^ progenitor cells while minimizing the risk of overtraining or injury. Long-term studies are also necessary to determine the effects of chronic exercise training on stem cell mobilization and tissue regeneration in athletes. Personalized exercise programs tailored to individual athletes' needs and considerations should be developed to optimize stem cell mobilization [[101], [102], [103]].

By harnessing the potential of exercise-induced stem cell mobilization, sports management professionals can enhance the well-being and performance of athletes. Future research endeavors in this field should prioritize the investigation of the impact of resistance exercise on populations of stem cells, with particular emphasis on optimizing variables that enhance precursor cell populations and ensuring the inclusion of women in these studies. To differentiate between the transient and sustainable benefits of stem cell mobilization through exercise, it is imperative to develop a more profound understanding of the underlying mechanisms involved, including the elucidation of molecular pathways, signaling mechanisms, and factors that regulate the functionality and longevity of mobilized stem cells. The standardization of measurement techniques and protocols assumes critical significance as it facilitates reliable comparisons across various studies, while the development of non-invasive and real-time monitoring techniques would provide valuable insights into the temporal dynamics of SC responses during exercise. Furthermore, exploring the correlation between exercise-induced changes in specific subsets of stem cells and the adaptability of tissues would contribute to determining the functional significance of SC mobilization. Long-term effects of exercise on stem cell populations should be thoroughly investigated to evaluate sustained impacts on tissue regeneration and overall health. Additionally, there is a pressing need to explore the therapeutic applications of exercise-induced stem cell mobilization, including interventions in specific disease populations and the assessment of exercise as an adjunct therapy.

## 5 Conclusion

The impact of exercise on SC populations is a significant and emerging area of scientific research. SCs play a critical role in tissue regeneration, repair processes, and maintaining physiological balance. Understanding how different types of exercise affect SC mobilization and function has the potential to develop exercise regimens aimed at enhancing overall health outcomes. This article has investigated the acute and chronic effects of aerobic and resistance exercise on SC populations in both healthy and diseased individuals, spanning various age groups. Research indicates that acute maximal exercise significantly contributes to the mobilization of precursor cells, with CD34^+^ subsets showing the highest responsiveness. However, studies on the effects of chronic exercise on CD34^+^ cells in circulation and their functionality have yielded variable results, likely due to differences in exercise dosage, individuals' health status, and the analytical methods used for cellular analysis. Even short-term exercise interventions lasting from 10 days to 3 weeks have been shown to enhance the mobilization of CD34+/KDR + angiogenic precursor cells in older adults, highlighting the body's rapid activation of reparative cell populations in response to physical activity. Several important avenues for future research emerge from the existing literature. While numerous studies have demonstrated an increase in the number of CD34^+^ cells with aerobic exercise, resistance exercise protocols have yet to be investigated. There is a pressing need for further research on optimizing resistance exercise variables that enhance precursor cell populations, particularly among women, who have received limited attention in this regard. Furthermore, a deeper mechanistic understanding is crucial for distinguishing between the transient and sustainable benefits of SC mobilization through exercise. Tracking how exercise-induced changes in CD34^+^ subsets correlate with vascular and tissue adaptability can provide valuable insights into the functional significance of cellular mobilization. Ultimately, further research should examine short-term exercise programs and evaluate their potential for providing the benefits of regeneration by temporarily activating the inherent population of SCs. In general, the current evidence confirms the integration of both acute intense activity and regular moderate aerobic exercise in exercise regimens to activate the potential for intrinsic body regeneration through SC mobilization. This article showed the effects of aerobic and resistance exercise on stem cells in healthy and diseased individuals of different ages. It found that both acute intense exercise and long-term moderate training can increase circulating precursor cell levels, particularly CD34^+^ subsets.

## Declaration of competing interest

The authors declare that they have no known competing financial interests or personal relationships that could have appeared to influence the work reported in this paper.
